# Co-Expression Analysis of the ZDHHC19 Palmitoyltransferase–miR-4733–miR-596 Putative Regulatory Axis in Sepsis

**DOI:** 10.3390/genes16040359

**Published:** 2025-03-21

**Authors:** Katalin Maricza, Zsuzsanna Elek, Eszter Losoncz, Krisztina Molnár, Zoltán Fülep, Réka Kovács-Nagy, Zsófia Bánlaki, Gergely Keszler, Zsolt Rónai

**Affiliations:** 1 Institute of Biochemistry and Molecular Biology, Department of Molecular Biology, Semmelweis University, 1085 Budapest, Hungary; maricza.katalin@stud.semmelweis.hu (K.M.); elek.zsuzsanna@semmelweis.hu (Z.E.); molnar.krisztina@stud.semmelweis.hu (K.M.); kovacs-nagy.reka@semmelweis.hu (R.K.-N.); banlaki.zsofia@semmelweis.hu (Z.B.); keszler.gergely@semmelweis.hu (G.K.); 2 Doctoral School, Semmelweis University, 1085 Budapest, Hungary; losoncz.eszter@phd.semmelweis.hu; 3 Department of Anesthesiology and Intensive Therapy, Bács-Kiskun County Teaching Hospital, 6000 Kecskemét, Hungary; fulepz@kmk.hu

**Keywords:** infection, sepsis, gene expression, microRNA, ZDHHC19, single nucleotide polymorphism

## Abstract

**Background**: ZDHHC19—a protein acyltransferase—is known to be induced in sepsis, a dysregulated immune response to infection, but the underlying molecular mechanisms remain elusive. In this study, we aimed to explore whether upregulation of *ZDHHC19* is modulated by single nucleotide polymorphisms (SNPs) affecting the binding of microRNA in the 3’ untranslated region of the gene. **Methods**: Inpatients with clinically verified severe infection (*n* = 83) or sepsis (*n* = 63) were recruited to the study. Genomic DNA and total RNA were prepared from buccal and peripheral blood samples, respectively. Genotyping of rs112579116 and rs2293161 SNPs was performed by TaqMan real-time PCR assays, while *ZDHHC19* mRNA as well as miR-4733 and -596 microRNA levels were quantitated by reverse transcription qPCR. Correlations between genotypes, expression levels and clinical parameters were assessed by the Shapiro–Wilk, Mann–Whitney and t-tests. **Results**: Transcript levels of *ZDHHC19* were significantly enhanced in septic blood samples (*p* = 0.0000709) and associated with clinical parameters such as procalcitonin levels, blood cell counts and clotting factors. Levels of both miRNAs showed an inverse but not significant correlation with those of ZDHHC19. **Conclusions**: Expression of ZDHHC19 should be considered a reliable molecular marker of sepsis, but further investigations are needed to shed light on regulatory mechanisms involved.

## 1. Introduction

According to the current definition, sepsis is a life-threatening organ dysfunction caused by a dysregulated immune response triggered by infection, which can progress to septic shock requiring vasopressor therapy [1]. This concise definition has supplanted earlier sepsis concepts (sepsis-1 and sepsis-2), which were based on the fulfillment of at least two SIRS (systemic inflammatory response syndrome) criteria and a confirmed or suspected infection. SIRS criteria include tachycardia (heart rate > 90 beats/min), tachypnea (respiratory rate > 20 breaths/min), fever or hypothermia (temperature > 38 °C or < 36 °C) and leukocytosis, leukopenia or bandemia (white blood cells > 1200/mm^3^, < 4000/mm^3^ or bandemia ≥ 10%) [2]. Despite early diagnosis, supportive therapies for multiple organs and antimicrobial treatments, sepsis remains a leading cause of death worldwide [3].

Although high-throughput transcriptomic [4] and proteomic studies [5] conducted on cells and even extracellular vesicles [6] have revealed extensive sepsis-specific alterations in gene expression patterns and molecular pathways, a reliable and universal molecular biomarker for early sepsis diagnosis is still urgently needed. Procalcitonin has long been used as a marker of infection and sepsis, and—although not routinely established in the clinical practice yet—a number of novel promising acute phase biomarkers for diagnosing and monitoring bacterial infection and sepsis are being evaluated, including presepsin, a cleavage product of CD14 and a soluble pattern recognition receptor [7], mid-regional pro-adrenomedullin (MR-proADM) [8] and neutrophil gelatinase-associated lipocalin [9]. While the importance of altered transcript and protein levels in the pathogenesis of sepsis is widely recognized, it is becoming increasingly clear that sepsis-specific modulation of protein functions via post-translational modifications might also play a role [10]. Notably, the epigenetic landscape in sepsis is shaped by several chromatin-modifying enzymes [11], and lactylation has recently been proposed as a potential molecular marker of sepsis [12], affecting both histones [13] and non-histone proteins [14].

S-palmitoylation is a relatively novel and intensively studied post-translational mod-ification in which long-chain fatty acids, most commonly palmitate (C16:0), are attached to cysteine residues of proteins via a thioester linkage [15]. In humans, S-palmitoylation is catalyzed by members of the zinc finger DHHC motif-containing ZDHHC enzyme family. Recently, protein S-palmitoylation, the only fully reversible lipid modification, has also been implicated in sepsis due to its versatility in fine-tuning virtually the entire spectrum of protein characteristics, including conformation, stability, localization, trafficking, activity and interactions [16]. Palmitoylation has been shown to activate key signal-transducing proteins in sepsis pathways, such as MYD88, which is essential for toll-like receptor (TLR) signaling upon lipopolysaccharide (LPS) stimulation in bacterial sepsis [17], and STING (stimulator of interferon genes), an immune-regulatory protein [18]. Conversely, inhibition of ZDHHC12 enhances the activity of the NLRP3 inflammasome and alleviates the symptoms of septic cardiomyopathy [19].

Among the 23 ZDHHC isoforms, ZDHHC19 has been reported to be induced in severe infections and under septic conditions, making it a potential sepsis biomarker. A fivefold upregulation of *ZDHHC19* expression has been observed in patients with acute respiratory distress syndrome (ARDS) [20]. *ZDHHC19* was also one of the genes upregulated in critically ill elderly septic patients on day 8 following diagnosis [21]. *ZDHHC19* is part of the 29-mRNA panel used in the InSep test to detect transcriptomic signatures characteristic of acute infection and sepsis. *ZDHHC19* mRNA turned out to be a robust marker of infection, and it was more profoundly induced in bacterial infections compared to viral ones [22]. Interestingly, ZDHHC19 seems to facilitate the course of some viral infections by palmitoylating key proteins indispensable for the pathogenesis: the spike protein of the SARS-CoV-2 virus [23], which may help the virus enter the cell, and the Chikungunya virus nsP1 protein [24] that enhances viral replication.

Recently, using an artificial neuronal network-based bioinformatics tool, Tong et al. identified eight key hub marker genes that seem to coordinate molecular pathways in sepsis, highlighting *ZDHHC19* as a principal linker gene connecting hubs marked by key genes such as *GPR84*, *CD177* and *TDRD9* [25]. The crucial role of ZDHHC19 in stabilizing signaling and metabolic networks in sepsis is particularly intriguing, given its broad range of substrates and interaction partners. Its principal substrates, R-Ras and STAT3 [26,27], are both involved in the proliferation and maturation of neutrophil granulocytes, which are key cellular mediators of immune responses. R-Ras is a well-known upstream activator of MAP cascades and has been shown to stimulate the phosphatidylinositol-3-kinase/protein kinase B/mTOR axis in neutrophils [28]. STAT3 is thought to play a key role in emergency granulopoiesis and neutrophil maturation, both of which are critical in sepsis [29,30].

Despite considerable progress in understanding the role of ZDHHC19 in sepsis, the molecular mechanisms underlying its induction remain elusive. This study aimed to confirm the molecular diagnostic value of *ZDHHC19* plasma levels for early sepsis diagnosis, disease progression monitoring, and severity assessment. Additionally, we explored potential post-transcriptional regulation of *ZDHHC19* by microRNAs (miRNAs), which are short, endogenous non-coding transcripts capable of binding target mRNAs to downregulate their expression. To this end, correlations were sought between transcript levels of *ZDHHC19* and two miRNA species potentially targeting it in plasma samples of patients with bacterial sepsis. Given that miRNAs bind in a sequence-specific manner, we also raised the question of whether common single nucleotide polymorphisms affecting miRNA binding sites might influence *ZDHHC19* levels and clinical parameters in sepsis.

## 2. Materials and Methods

### 2.1. Participants

A total of 146 patients were included in the study. They were admitted to one of the following hospital departments in Hungary: Bács-Kiskun County Teaching Hospital in Kecskemét (Department of Emergency Medicine); Semmelweis University in Budapest (Department of Emergency Medicine); and the Péterfy Sándor Street Hospital and Outpatient Clinic in Budapest (Department of Anaesthesiology and Intensive Therapy).

All participants were over the age of 18. Sample collection was performed after obtaining written informed consent from the patients. Ethics approval was granted by the Semmelweis University Regional and Institutional Committee of Science and Research Ethics (permission No.: 39/20j14) and by the National Centre for Public Health and Pharmacy, Department of Clinical Research (permission No.: NNGYK/GYSZ/10177-5/2024). This study was conducted in accordance with the WMA Declaration of Helsinki, and the handling of patient data was in compliance with the General Data Protection Regulation (GDPR) of the European Union.

Only patients with acute, new-onset infection were included in the study, while those with recurrent or chronic infection were excluded. Further exclusion criteria were malignant, autoimmune, metabolic or inflammatory diseases as well as immunosuppressive therapy. All participants suffered from a bacterial or viral infection confirmed through physical examination, laboratory tests, medical imaging (X-ray and computer tomography) and microbiological findings. The presence of SARS-CoV-19 infection was detected by rapid antigen tests and corroborated by standard reverse transcription qPCR assays from nasal swabs. If the anamnesis, patient complaints, physical examination, imaging and laboratory test results suggested a bacterial infection, microbiological culturing and empirical antibiotic therapy were initiated, which were later adjusted if necessary in light of the culture results and antibiogram. The following pathogens were successfully cultured from blood, urine samples and/or abdominal fluid samples: *Clostridium perfringens*, *Citrobacter koseri*, *Escherichia coli*, *Klebsiella pneumoniae*, *Prevotella buccae* et *disiens*, *Pseudomonas aeruginosa*, *Staphylococcus aureus*, *Streptococcus anginosus* and *salivarius*. The cutoff value of procalcitonine was 0.5 ng/mL, but this marker was primarily used to monitor the effectiveness of therapy.

Patients were classified into the septic cohort according to the latest international recommendations [1] with qSOFA scores ≥ 2. The qSOFA (quick Sequential (Sepsis-related) Organ Failure Assessment) scoring system can be utilized alongside either a high clinical suspicion or a confirmed infectious condition to rapidly and effectively identify patients with suspected sepsis. It incorporates three criteria, with each fulfilled criterion contributing one point to the score. Thus, the total score ranges from 0 to 3, and a score of 2 or 3 indicates a high suspicion of sepsis. The criteria are as follows: (i) respiratory rate ≥ 22 breaths/minute; (ii) systolic blood pressure ≤ 100 Hgmm; and (iii) altered mental status (Glasgow Coma Scale [GCS] < 15) [2]. 

All participants were Hungarian citizens of Caucasian origin.

### 2.2. DNA Sampling and Purification

Buccal epithelial cells were collected using swabs in duplicates (two swabs per sample and two samples per person). To purify genomic DNA, buccal samples were incubated in 0.2 mg/mL proteinase K cell lysis buffer at 56 °C overnight. Proteins were then precipitated with a saturated NaCl solution and removed by centrifugation. DNA was isolated from the supernatant using the standard ethanol/isopropanol precipitation method. The DNA pellet was resuspended in 100 µL of 0.5× TE buffer (0.005 M Tris-HCl, pH = 8 and 0.5 mM Na_2_EDTA). All reagents were purchased from Merck (Darmstadt. Germany) DNA concentration and purity were assessed using a NanoDrop 1000 spectrophotometer (Thermo Fisher Scientific, Waltham, MA, USA). The average DNA concentration of the samples was 123 ng/µL (range: 25–582 ng/µL).

### 2.3. Blood Sampling, RNA Extraction and cDNA Synthesis

Peripheral blood samples from patients were collected into Tempus™ Blood RNA Tubes obtained from Thermo Fisher Scientific. According to the protocol, the collecting tubes were stored at room temperature for no longer than 5 days and then at −20 °C until further processing. Total RNA was isolated using the MagMax™ for Stabilized Blood Tubes RNA Isolation Kit (Thermo Fisher Scientific). The average RNA concentration of the samples was 268 ng/µL (range: 20–1005 ng/µL). Following electrophoretic quality control, RNA was reverse transcribed into cDNA using the SuperScript™ VILO™ cDNA Synthesis Kit (ThermoFisher Scientific) and the miRNA 1st-Strand cDNA Synthesis Kit (Agilent Technologies, Santa Clara, CA, USA).

### 2.4. Human Tissue RNAs

Human tissue RNAs were obtained from Agilent Technologies (Santa Clara, CA, USA).

### 2.5. Quantitative Real-Time PCR Assays

Expression levels of *ZDHHC19* were measured using predesigned TaqMan gene expression assays. *GAPDH* was used as the internal reference gene (Hs00376116_m1 and Hs99999905_m1, respectively, from Thermo Fisher Scientific). For validation, 10% of the samples were randomly reanalyzed, confirming the initial results in 98.92% of cases. The High-Specificity miRNA qPCR Core Reagent Kit (Agilent Technologies) was used for miRNA expression analysis. The specific forward primer sequences for each miRNA are listed in Table 1.

### 2.6. NormFinder Analysis

The optimal reference gene for miRNA quantification was determined using NormFinder, a statistical algorithm that ranks candidate genes based on expression stability. It evaluates expression stability within a given group and analyzes the variability within and between these groups [31,32]. Briefly, the application calculates the mean expression of the genes for the given group and measures the deviation of each sample from this average. Intra-group variance is determined from the standard deviation of a given gene, which characterizes the variability of gene expression within each group. The top-ranked gene with the smallest stability value is considered the most stably expressed gene in the sample set. To examine the variability of gene expression between different groups, the average values for the given gene are compared, and the inter-group variance is calculated. This parameter reflects the stability of expression levels under different conditions. The stability index is then derived by summing the intra- and inter-group variance values. Potential control genes are ranked based on this index [31].

### 2.7. Statistical Analysis

Statistical analyses were performed using SPSS software (version 28.0.1.0). The Shapiro–Wilk test was applied to assess normality of a distribution. For normally distributed data, an independent sample t-test was used for group comparisons. For non-normally distributed data, the Mann–Whitney U test and Kruskal–Wallis test were used. For normally distributed data, an independent sample t-test was used to compare the groups, while the Mann–Whitney U test was used for non-normal distributions. Spearman’s correlation was applied to assess relationships between gene expression and continuous clinical parameters. To account for false positive results, an online adjustment tool for multiple correction testing was used (https://multipletesting.com, accessed on 19 August 2024), enabling false discovery rate (FDR) analysis [33]. Genotype frequencies were analyzed using the chi-square test, with all reported distributions conforming to Hardy–Weinberg equilibrium (*p* > 0.05).

## 3. Results

### 3.1. In Silico Analysis of miRNA Binding

**Figure 1:**
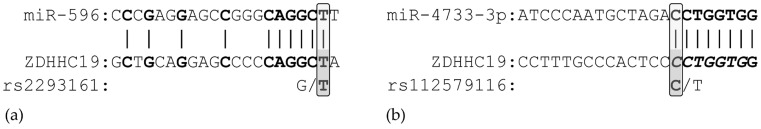
In silico sequence alignment of candidate microRNAs to the 3’-UTR of the *ZDHHC19* gene. miRNA nucleotides complementary to the target sequence are printed in bold. Major and minor alleles of SNPs affecting the seed region are shown against a gray background. Sequence alignment is shown for miR-596 (**a**) and for miR-4733-3p (**b**), respectively, in this figure.

To investigate the potential role of microRNAs in the regulation of ZDHHC19 expression in sepsis, we first needed a comprehensive list of miRNAs that potentially bind to the gene’s mRNA. To this end, the miRWalk v3 database was employed, which predicted a total of 1288 different miRNAs capable of binding to the *ZDHHC19* mRNA at 1928 different positions ([34], accessed on 30 May 2024). As the effect of miRNA on gene expression does not solely depend on the molar ratio of mRNA and miRNA but might also be influenced by single nucleotide variations termed miR-SNPs that affect the seed region, we were prompted to check whether any such polymorphisms exist in the *ZDHHC19* gene. After consulting the PolymiRTS v3.0 database ([35], accessed on 31 May 24), it turned out that the rs112579116 G/T and rs2293161 C/T single nucleotide polymorphisms might alter the binding sites of miR-4733-3p and miR-596, respectively. As schematically shown in Figure 1, the minor T allele of the rs2293161 SNP and the major C allele of the rs112579116 SNP create complementary seed regions, while the other alleles abrogate miRNA binding.

These in silico results raised the intriguing possibility that the expression of ZDHHC19 might be controlled by either or both miRNAs in a genotype-dependent fashion. To investigate this issue under in vivo conditions, we examined whether plasma levels of *ZDHHC19* mRNA and the two regulatory miRNAs change in a coordinated manner in patients with sepsis and whether there is a correlation between the genotypes of both miR-SNPs and gene expression, as well as the clinical progression of sepsis.

### 3.2. Gene Expression Analysis in Healthy Human Tissue Samples

Co-expression of a microRNA and its target gene is an obvious prerequisite for microRNA-mediated gene expression regulation. However, there is limited information in the literature regarding whether *ZDHHC19* and its bona fide regulatory microRNAs are expressed in the same tissue, and the available data are inconsistent due to different sampling and processing methods. To clarify this, total RNA was isolated from 13 different human tissue samples, and expression levels of *ZDHHC19* and our candidate microRNAs were quantitated by reverse transcription–quantitative polymerase chain reaction (RT-qPCR).

Careful selection of a proper reference gene(s) with relatively stable expression levels in the biological sample is a cornerstone of relative quantification and a token of reliability in transcriptomic studies. As per standard practice, researchers deploy different reference transcripts for quantification of long and short RNA species to ensure optimal performance under the given experimental conditions. Of the housekeeping gene set commonly used in the literature, we determined the transcript levels of hypoxanthine guanine phosphoribosyl-transferase 1 (*HPRT1*) and glyceraldehyde-3-phosphate dehydrogenase (*GAPDH*) gene as putative candidates for *ZDHHC19* quantification and those of *U6* small nuclear RNA as the control for miRNA expression. Subsequently, the NormFinder stability analyzer software was used to calculate a stability index (ρ) for each transcript. The lowest index marks the most suitable reference gene with the most stable expression levels across samples.

**Figure 2:**
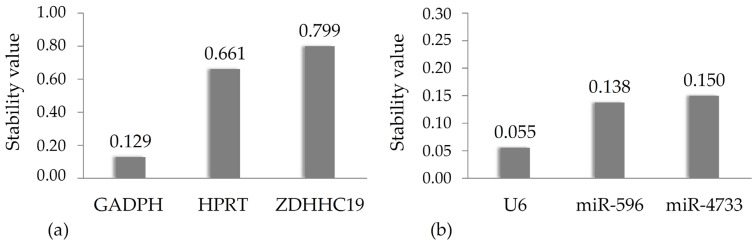
Representation of gene expression stability in the blood sample dataset as calculated with NormFinder. The overall stability index (ρ) is shown for each gene in increasing order. Panel (**a**) shows data for mRNAs while panel (**b**) shows data for *U6* and both miRNAs.

Stability indices are displayed in Figure 2 for mRNA (a) and miRNA (b), respectively. As the stability indices of *GAPDH* (ρ = 0.129) and *U6* (ρ = 0.055) are sufficiently low, they were used for relative quantification throughout our experiments.

**Figure 3:**
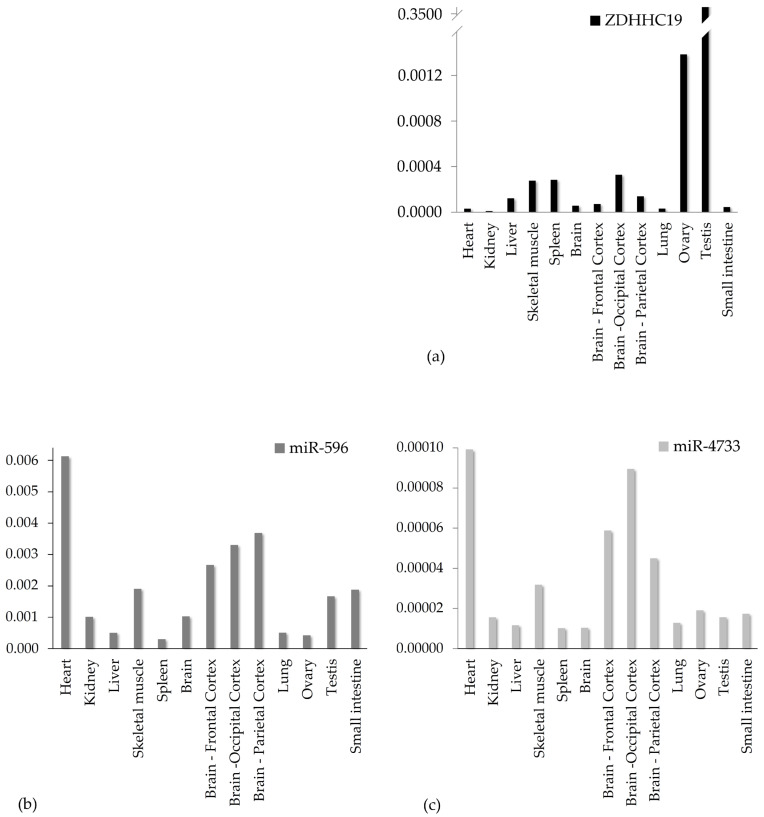
Relative transcript levels in different normal human tissues. Relative expression of *ZDHHC19* is shown in panel (**a**); that of miR-596 in panel (**b**) and that of miR-4733-3p in panel (**c**). *ZDHHC19* transcript levels were normalized to *GAPDH*, while those of both microRNAs were normalized to *U6*.

Normalized, tissue-specific transcript levels for *ZDHHC19* (a), miR-596 (b) and miR-4733-3p (c) are shown in Figure 3. In agreement with the literature, *ZDHHC19* exhibited by far the highest expression in the testes. Below this level, two orders of magnitude lower, the ovary followed, while in the other examined tissues, much lower mRNA levels were found, at times barely detectable. Importantly, no such large expression differences were observed with regard to the miRNAs. Both miRNAs were detectable in all tissues, although miR-4733-3p was present in much lower amounts than miR-596. Surprisingly, however, the expression patterns of the two miRNAs were highly similar: higher than average levels were detected in the heart and skeletal muscle, as well as in the neocortex, implicating some common mechanisms regulating their expression. Assuming that miRNAs downregulate target gene expression, there seems to be a strong inverse correlation between ZDHHC19 and miRNA levels. For instance, high gonadal expression of ZDHHC19 contrasts with very low levels of both miRNAs, while in the skeletal muscle, where both miRNAs are highly expressed, ZDHHC19 transcripts are barely detectable.

In summary, ZDHHC19 and its putative regulatory microRNAs are expressed in all tissues examined, and inverse correlations between transcript levels imply that these miRNAs may indeed play a role in fine-tuning ZDHHC19 expression.

### 3.3. Characterization of Patient Cohorts by Clinical Parameters

Patients recruited for the study were classified into ‘infected’ and ‘septic’ groups using the qSOFA scoring system, which is widely used in clinical practice; patients were ordered into the septic group if they had qSOFA scores ≥ 2 [1]. Blood samples were taken when the diagnosis of an underlying infection was set up and were subsequently divided into ‘infected’ or ‘septic’ groups in light of qSOFA scoring. Therefore, blood samples correspond to an early, untreated phase of severe infection or sepsis. Both infected and septic cohorts were stratified further according to the localization of the primary infection. The most populous subgroups included cases of respiratory, urogenital and abdominal origin (Table 2). All patients were tested for Covid-19 infection.

To better characterize these clinical cohorts, the PIRO concept was implemented, offering a more comprehensive way to sepsis detection and staging [36]. This concept considers not only the presence of predisposing factors but closely monitors the course of the inflammatory response to infection and organ dysfunction by a detailed analysis of clinical parameters. As shown in Table 2, significantly worse inflammatory (CRP, PCT), respiratory (respiratory rate, SpO2), cardiovascular (systolic and diastolic pressure, MAP, heart rate, shock index), hepatic (ASAT, ALAT, GGT, LDH), renal (crea, CN) and clotting (INR, D-dimer) parameters were observed in the septic group. Furthermore, patients in the septic group required various organ function replacement and supportive treatments, while those in the infected group did not. These results corroborate the diagnosis of sepsis previously established using qSOFA scores.

### 3.4. Gene Expression Analysis in the Patient Cohorts

**Figure 4:**
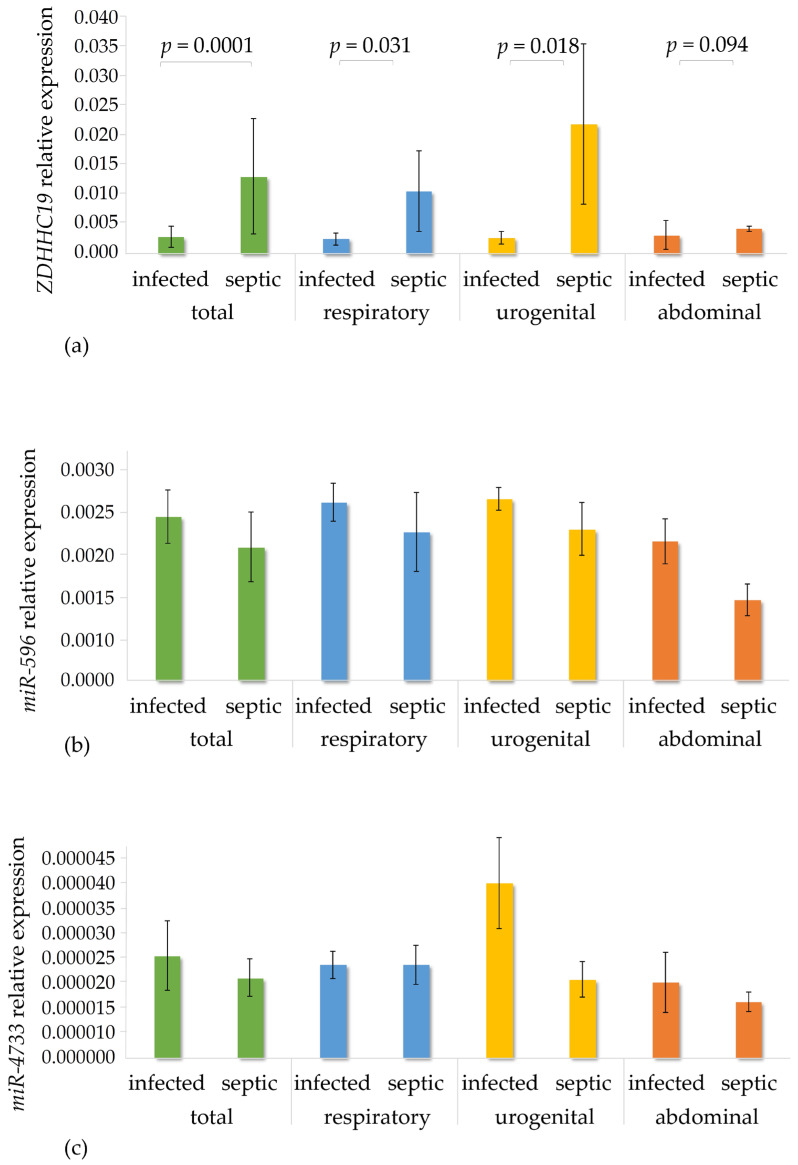
Bars depict average relative expression levels of *ZDHHC19* (**a**), miR-596 (**b**) and miR-4733-3p (**c**) in the total infection and sepsis groups as well as in sub-cohorts stratified according to origin of infection. Statistical comparisons between the groups were performed using the Mann–Whitney test, with *p*-values displayed above the bar charts.

Earlier studies provided clear-cut evidence that plasma levels of *ZDHHC19* are elevated in sepsis, but there is scarce information on factors responsible for upregulation of ZDHHC19 expression at the transcriptional and post-transcriptional levels. To confirm these data in our patient cohorts and to find correlations between transcript levels of *ZDHHC19* and both of its putative regulatory microRNAs, total RNA was prepared from plasma samples, and transcript levels were quantified by real-time PCR assays and normalized to reference genes, as described in Section 3.2. Transcript levels of *ZDHHC19*, miR-596 and miR-4733-3p in the infection and sepsis populations as well as in the septic subgroups are shown in panels a, b and c of Figure 4, respectively.

Significant differences were found in relative expression levels of *ZDHHC19* between the infected and the total septic cohorts as well as between respiratory and urogenital septic sub-cohorts, while transcript levels were slightly but not significantly elevated in the abdominal group. Induction of our target gene was most profound in sepsis of urogenital origin. Importantly, expression of both miRNAs was lower in the total septic group and all subgroups as compared to the infected group. Nevertheless, an inverse correlation between mRNA and miRNA levels implies that upregulation of *ZDHHC19* transcript levels might partly be attributed to reduced miR-4733-3p and miR-592 expression in sepsis. It is quite intriguing that a more than twofold downregulation of miR-4733-3p expression was observed in patients with urogenital sepsis, the cohort with the most profound induction of *ZDHHC19*.

Based on data presented in Figure 4, following a reverse approach, we also aimed to find out whether the origin of sepsis (abdominal, respiratory or urogenital) determines ZDHHC19 and miRNA expression levels. To assess this, transcript levels in the three septic subgroups were compared systematically to those in the corresponding infection sub-cohorts using the Kruskal–Wallis test. Since no significant differences were found in any comparison, it can be concluded that the origin of infection does not exert significant influence on gene expression signatures.

Next, we examined whether there was statistical correlation between any clinical parameter associated with sepsis presented in Table 2 and transcript levels of ZDHHC19 and both miRNAs in the sepsis group. As can be seen in Table 3, expression levels of ZDHHC19 were significantly associated with procalcitonin levels, neutrophil, lymphocyte and platelet counts and with the INR coagulation parameter. On the other hand, the only parameter associated with miR-596 was the level of D-dimers, while miR-4733-3p did not show any significant association with the clinical parameters monitored.

### 3.5. Genotyping and Association Analyses

Both miR-SNPs (rs112579116 and rs2293161) localized within the 3’ untranslated region (UTR) of the *ZDHHC19* gene were genotyped in the infected and septic cohorts by running pre-designed genotyping TaqMan probe-based qPCR assays. Table 4 (A) presents the locations, allele frequencies and genotype distribution of both polymorphisms, which are in Hardy–Weinberg equilibrium (*p* > 0.05).

Both polymorphisms were subjected to linkage disequilibrium (LD) analysis. Our results are consistent with those obtained from the 1000 Genomes Project dataset. The level of LD was notably low (D’ = 2; R^2^ = 0), indicating the independent inheritance of both loci. Therefore, both SNPs were included in the subsequent association analysis.

Allele frequencies obtained by TaqMan probe-based genotyping are shown in Table 4 (B). Statistical analysis, however, revealed that they did not differ significantly between the infection and sepsis groups.

Next, we raised the question of whether the allele frequencies of both polymorphisms are associated with infection or sepsis, but no significant association was found, and neither SNP was found to be associated with the respiratory, urogenital and gastrointestinal sub-cohorts of the sepsis group (Table 5). No associations were found between allele frequencies of both miR-SNPs and clinical parameters presented in Table 2 either.

Since the SNP alleles did not show any association with any disease or cohort individually, we were prompted to seek potential associations between haplotype combinations and the cohorts. As shown in Table 6, haplotype frequencies obtained from our sample did not differ significantly from those of the 1000 Genomes Project. Only three of the four possible haplotypes were present in our sample, of which GC had by far the highest frequency. Although the GC haplotype was more common, while the GA haplotype was less frequent in the septic than in the infection group, these differences did not reach statistical significance.

## 4. Discussion

Sepsis continues to be a frequently fatal complication of infection, presenting with dramatically altered transcriptomic, proteomic and metabolomic landscapes in affected tissues [37,38]. In light of this, several studies have been launched to identify early biomarkers of sepsis with strong predictive value. The objectives of these studies vary widely, ranging from the identification of individual markers [39] to complex omics approaches, where an entire panel of molecular fingerprints is examined simultaneously [40]. Given the sequence of gene expression phases, it is undeniable that transcriptomic changes respond the most rapidly to the deterioration of the infected patient’s condition. With the rapid development of high-throughput transcript quantitation assays in recent years, transcript analysis in sepsis has expanded to include both long [41] and short [42] non-coding RNAs. Several studies have identified microRNAs as sensitive and specific biomarkers, including miR-34a-5p and miR-199a-3p in neonatal sepsis [43], as well as miR-486-5p [44] and miR-10a [45], among others, whose levels may correlate with sepsis severity and patient outcomes.

As early and specific antimicrobial therapy is the cornerstone of sepsis treatment, it is essential to distinguish SIRS from sepsis triggered by viral or bacterial pathogens. Recent findings suggest that analyzing transcript host-RNA signatures (reviewed in [46]) may help identify the origin of the infection, offering invaluable support in selecting the appropriate antimicrobial medication.

Having previously investigated transcriptomic changes in signaling pathways in sepsis [47], the purpose of our present study was to analyze co-expression of members of a complex posttranscriptional regulatory network comprising two microRNAs and their putative target, *ZDHHC19*, a gene known to be induced in sepsis. This network-centered approach is becoming increasingly recognized for providing a more in-depth insight into the pathomechanism of the disease compared to studies that focus solely on individual transcripts, and parallel changes in transcript levels in these networks are considered more reliable progression markers [48]. Using bioinformatics tools, the power and scope of these co-regulatory–co-expression assays in sepsis can be vastly extended [49].

Using in silico tools, binding of miR-4733-3p and miR-596 to the 3’ untranslated region (UTR) of *ZDHHC19* was predicted to be modulated by rs112579116 (G/T) and rs2293161 (C/T) miR-SNPs, suggesting that the expression of *ZDHHC19* could be regulated by these miRNAs in a genotype-dependent manner. Although the PolymiRTS database suggests that additional microRNAs may bind to the above-mentioned polymorphisms in the 3’-UTR, literature data were only available for miR-4733-3p and miR-596, which is why we chose to focus on them. To the best of our knowledge, however, neither of these miRNAs has been studied in relation to infection or sepsis. However, their role in regulating cancer cell proliferation and survival has already been established. Overexpression of miR-596 in melanoma effectively inhibited the MAPK/Erk signaling pathway by downregulating *MEK1*, *MCL1* and *BCL2L1* levels [50]. Additionally, it could reduce cell growth and motility through the Wnt/β-catenin signaling pathway in prostate cancer [51] and in the metastases of non-small-cell lung cancer as well [52]. MiR-4733, on the contrary, seems to have tumor-promoting properties in gall bladder cancer cells, as it directly suppressed expression of KLF7, an anti-oncogene, and E-cadherin, a key player in the epithelial-mesenchymal transition process [53].

In our study, co-expression of *ZDHHC19* and both miRNAs was assessed under both physiological and pathological conditions.

First, RT-qPCR assays run on a series of healthy human tissue samples revealed that *ZDHHC19* was predominantly expressed in the testes, with lower expression levels in ot-her tissues such as the ovary and skeletal muscle. In good agreement with our results, Ohono et al. reported high levels of *ZDHHC19* in the testes and weak expression in the thymus and small intestine [54]. According to the Human Tissue Atlas (https://www.proteinatlas.org/ENSG00000163958-ZDHHC19/tissue, accessed on 21 March 2024), in addition to the testis, mild expression was seen in the spleen and adrenal gland, while data in the GTEx Transcript Browser (https://www.gtexportal.org/home/transcriptPage; accessed on 21 March 2024) reveal high expression in the testes, ovaries, spleen, adrenal gland and whole blood.

Currently, there is no consensus on which miRNA should be used as a reference in relative quantitation qPCR assays. *U6* is perhaps one of the most widely used endogenous controls in miRNA studies across a variety of tissue types and clinical conditions [55,56], including miRNA assays from the whole blood of the septic patients we have studied [57,58]. However, when the stability of *U6* has been tested against other potential reference genes, conflicting results have been obtained. Some authors have found its expression to be stable [59], while others have found it insufficiently stable and therefore not suitable for use as an endogenous control [60]. Consequently, we tested its stability with the NormFinder software, and it turned out to be a suitable endogenous control in our samples.

We consider it important to highlight that while miR-4733-3p and miR-596 were detectable in all the examined tissues, their concentrations showed an inverse correlation with the *ZDHHC19* levels in most of the tissues. This may be an indirect indication that these two miRNAs might indeed regulate the expression of *ZDHHC19*.

While most sepsis studies use healthy individuals as a control group, we compared gene expression levels in sepsis with those in infection. This approach enabled us to identify more fine-tuned, sepsis-associated molecular signatures, better highlighting the pivotal point when immune responses get dysregulated and septic conditions develop [47].

In patients with sepsis, *ZDHHC19* expression was significantly elevated compared to the infection group, especially in urogenital sepsis. Interestingly, expression levels of miR-4733-3p and miR-596 were lower in the total septic cohort as well as in its origin-related sub-cohorts compared to the corresponding infection cohorts, supporting the hypothesis, which was also observed in healthy samples, that reduced miRNA expression might contribute to *ZDHHC19* upregulation during sepsis. The most striking—though not significant—downregulation was observed for miR-4733-3p in urogenital sepsis, where *ZDHHC19* expression was most pronounced. This finding suggests that miR-4733-3p may have a particularly crucial role in regulating *ZDHHC19*. However, further studies conducted on larger patient populations are required to confer statistical significance on these interactions and assess their functional significance.

Additionally, our association analysis highlighted significant correlations between *ZDHHC19* expression and clinical parameters such as procalcitonin, neutrophil count and INR, reinforcing the role of *ZDHHC19* in the systemic response to infection. The miRNAs, however, did not show consistent associations with clinical parameters, which could indicate that their regulatory effects are more subtle or context-dependent, requiring further exploration.

Contrary to our expectations, however, the genotyping assays revealed no significant differences in the allele and haplotype frequencies of the rs112579116 and rs2293161 SNPs between the infected and septic groups, and no association was found between allele frequencies and any clinical parameters either. This suggests that while these SNPs may alter miRNA binding, their subtle, direct impact on *ZDHHC19* expression in sepsis is likely overshadowed by stronger molecular factors mediating inflammatory responses and organ dysfunction. For instance, *ZDHHC19* expression in sepsis should primarily be up-regulated at the transcriptional level. According to the GeneCard Database (https://www.genecards.org/cgi-bin/carddisp.pl?gene=ZDHHC19#expression, accessed on 21 March 2024), major transcription factors predicted to bind to the *ZDHHC19* promoter include C/EBPbeta, CREB, deltaCREB, E47, FOXO4, HOXA5, Lmo2, NRSF form 1 and USF-1, and some of these are reportedly modulated under septic conditions [61,62,63]. On the other hand, although miR-SNPs in the seed region of miRNA binding usually do affect the base pairing between microRNAs and mRNAs, in some cases, the change in the affinity is not significant enough to prevent binding. Moreover, mRNAs may contain multiple binding sites, and the miR-SNP might not affect the most important of them. These open questions should be clarified by means of reporter assays performed in transient transfection experiments.

Overall, this study provides experimental evidence that miRNAs, particularly miR-4733-3p and miR-596, are involved in the regulation of *ZDHHC19* in sepsis. The inverse relationship between ZDHHC19 and miRNA expression suggests that dysregulation of miRNA levels may contribute to the pathological upregulation of *ZDHHC19* in sepsis, providing a potential avenue for therapeutic interventions aimed at modulating miRNA expression to control ZDHHC19 levels.

## Figures and Tables

**Table 1:** The sequences of specific primers used to measure microRNA expression.

miRNA	Sequence
miR-4733-3p	5′ CCACCAGGTCTAGCATTGGGAT 3′
miR-596	5′ AAGCCTGCCCGGCTCCTCGGG 3′
miR-4293	5′ CAGCCTGACAGGAACAG 3′
miR-6078	5′ CCGCCTGAGCTAGCTGTGG 3′
U6	5′ TTCGTGAAGCGTTCCATATTTT 3′

**Table 2:** Clinical and biological characterization of subject cohorts. Standard deviations (where relevant) are indicated. The comparison of medians between infected and septic groups was performed using the Mann–Whitney U test, the results of which are shown in the *p*-value column of the table. *p*-values that remained statistically significant after multiple correction are printed in bold. Abbreviations: ALAT, alanine aminotransferase; ALP, alkaline phosphatase; APTT, activated partial thromboplastin clotting time; ASAT, aspartate aminotransferase; AVP, arginine vasopressin; CN, carbamide; Crea, creatinine; CRP, C-reactive protein; ECMO, extracorporeal membrane oxygenation therapy; GGT, γ-glutamyl transferase; Hgb, hemoglobin; Htc; hematocrit; INR, international normalized ratio; IRRT/CRRT, intermittent/continuous renal replacement therapy; IPPV; invasive positive pressure ventilation; LDH, lactate dehydrogenase; MAP, mean arterial pressure; NIV, non-invasive ventilation; PCT, procalcitonin; PLT, platelet count; RBC, red blood cell count; SpO_2_, oxygen saturation; WBC, white blood cell count.

	Infection	Sepsis	*p*-Value
**Demographics**			
Number of patients	83	63	
mean age (years)	44.2 ± 14.0	51.1 ± 12.6	**5.50 × 10^−3^**
ratio of males (%)	60.2	73.0	1.17 × 10^−1^
**Origin of infection**			
Abdominal (%)	26.5	4.8	**1.86 × 10^−3^**
Urogenital (%)	13.3	12.7	1.00 × 10^0^
Respiratory (%)	49.4	81.0	**5.38 × 10^−5^**
**Type of infection**			
Viral (SARS-CoV-19) (%)	11.1	27.0	
Bacterial (%)	88.9	73.0	
Inflammation			
CRP (mg/L)	90.5 ± 87.7	192.0 ± 127.2	**1.00 × 10^−7^**
PCT (µg/L)	1.2 ± 7.2	20.5 ± 64.5	**6.20 × 10^−7^**
**Hemogram**			
RBC (× 10^12^/L)	4.8 ± 0.6	4.5 ± 0.9	8.81 × 10^−2^
Hgb (g/L)	140.1 ± 18.9	137.5 ± 19.7	3.88 × 10^−1^
Htc (%)	23.1 ± 20.6	10.3 ± 24.2	**2.33 × 10^−5^**
WBC (× 10^9^/L)	11.4 ± 5.7	11.7 ± 6.9	9.30 × 10^−1^
neutrophils (%)	75.4 ± 12.4	84.7 ± 8.7	**1.65 × 10^−8^**
lymphocytes (%)	15.3 ± 8.1	9.9 ± 7.6	**1.10 × 10^−5^**
PLT (× 10^9^/L)	267.8 ± 111.6	237.8 ± 123.0	8.93 × 10^−2^
**Coagulation**			
INR	1.2 ± 0.2	1.4 ± 0.3	**4.19 × 10^−3^**
D-dimer (μg/mL)	1.7 ± 3.7	7.0 ± 14.7	**8.2 × 10^−4^**
APTT (s)	35.2 ± 10.1	37.2 ± 12.1	6.57 × 10^−1^
fibrinogen (g/L)	5.5 ± 2.1	8.2 ± 5.6	**1.53 × 10^−2^**
**Cardiovascular function**			
Systolic pressure (Hgmm)	128.7 ± 20.0	118.2 ± 29.8	**1.86 × 10^−2^**
Diastolic pressure (Hgmm)	82.7 ± 11.1	71.8 ± 17.7	**3.24 × 10^−4^**
MAP (Hgmm)	98.0 ± 13.4	90.5 ± 23.7	4.05 × 10^−2^
Heart rate (1/min)	98.6 ± 17.0	105.3 ± 19.2	2.57 × 10^−2^
Shock Index	0.8 ± 0.2	1.0 ± 0.4	**5.21 × 10^−3^**
Norepinephrin (%)	0	41.7	-
AVP (%)	0	5.3	-
Dobutamine (%)	0	1.7	-
Dopamine (%)	0	3.4	-
**Respiratory function**			
SpO_2_ (%)	96.2 ± 4.2	83.1 ± 14.0	**3.66 × 10^−10^**
Respiratory rate (1/min)	17.1 ± 3.5	28.2 ± 7.2	**1.55 × 10^−15^**
High flow O_2_ therapy (%)	1.2	8.6	-
NIV (%)	0	6.9	-
IPPV (%)	0	46.6	-
ECMO (%)	0	5.2	-
Treatment in hospital (days)	7.9 ± 6.4	18.6 ± 19.4	**9.76 × 10^−8^**
Treatment in ICU (days)	0.3 ± 1.2	6.1 ± 10.7	**1.28 × 10^−7^**
Mortality (%)	0	23.2	**6.63 × 10^−9^**
**Liver function**			
ASAT (IU/L)	44.4 ± 51.0	111.7 ± 367.5	**8.21 × 10^−6^**
ALAT (IU/L)	38.9 ± 32.1	95.0 ± 304.8	**5.93 × 10^−3^**
GGT (IU/L)	87.1 ± 172.3	118.6 ± 107.8	**2.30 × 10^−4^**
ALP (IU/L)	102.7 ± 141.9	102.8 ± 72.2	2.47 × 10^−1^
Total bilirubin (µmol/L)	9.8 ± 5.6	15.6 ± 24.0	9.42 × 10^−2^
LDH (IU/L)	481.2 ± 828.3	526.0 ± 276.0	**2.64 × 10^−3^**
**Renal function**			
Crea (µmol/L)	81.1 ± 28.6	138.3 ± 153.0	**1.45 × 10^−3^**
CN (mmol/L)	6.4 ± 11.0	9.9 ± 10.1	**4.52 × 10^−6^**
serum Na^+^ (mmol/L)	137.9 ± 2.7	136.4 ± 5.3	1.09 × 10^−1^
serum K^+^ (mmol/L)	4.1 ± 0.6	4.2 ± 0.5	2.35 × 10^−1^
IRRT/CRRT (%)	0	17.2	-

**Table 3:** Association analysis of expressions levels and clinical characteristics of septic patients. Gray backgrounds show nominally significant results; bold numbers are significant using FDR approach for multiple testing. For significant results, the correlation coefficient (ρ) and the number of samples (N) are also indicated (*p*: level of significance).

	Procalcitonin	Neutrophil	Lymphocyte	Platelet	INR	D-Dimer
Expression of ZDHHC19	*ρ* = 0.380 *p* = 0.029 *N* = 33	*ρ* = 0.449 *p* = **0.0004** *N* = 58	*ρ* = −0.410 *p* = **0.0014** *N* = 58	*ρ* = −0.444 *p* = **0.0005** *N* = 57	*ρ* = 0.421 *p* = 0.0229 *N* = 29	*p* = 0.1939
Expression of miR-596	*p* = 0.1005	*p* = 0.0516	*p* = 0.0538	*p* = 0.1462	*p* = 0.2863	*ρ* = −0.657 *p* = 0.0202 *N* = 12
Expression of miR-4733-3p	*p* = 0.6287	*p* = 0.6840	*p* = 0.3909	*p* = 0.5584	*p* = 0.9182	*p* = 0.9656

**Table 4:** (A). Characterization of both single nucleotide polymorphisms (SNPs) genotyped in this study. SNPs are listed in the order of their genomic location taken from the Genome Reference Consortium human 38 (GRCh38) database. Literary minor allele frequencies (MAF) are from the gnomAD browser database (https://gnomad.broadinstitute.org, accessed on 4 April 2024) and correspond to data obtained in the European population. Levels of statistical significance for Hardy–Weinberg equilibrium (HWE) were calculated using the *χ*^2^-test. (B). Allele frequencies in the infection and sepsis cohorts as determined by qPCR-based genotyping.

(A)						
SNP	Allele	Genomic Location (GRCh38.p14)	Intragenic location	MAF	TaqMan ID	HWE (*p*)
rs112579116	C/T	chr3:196197507	3’ UTR	0.07 (T)	C__99261213_10	0.85
rs2293161	G/T	chr3:196197677	3’ UTR	0.04 (T)	C__15970299_10	0.36
**(B** **)**						
**SNP**		**Allele**	**Allele Frequency (%)**			***p*-Value**
			**Infection Cohort**		**Sepsis Cohort**	
rs112579116		C	97.3		85	0.37
		T	2.7		3	
rs2293161		G	95.9		80	0.19
		T	4		8	

**Table 5:** Association analysis between single nucleotide polymorphisms in the *ZDHHC19* gene and the presence of infection, sepsis or a sepsis subgroup. *p*-values are shown.

SNP	Infection vs. Sepsis	Sepsis From		
		Respiratory	Urogenital	Abdominal
rs112579116	0.7530	0.3007	0.3126	0.8681
rs2293161	0.0660	0.2380	0.8450	0.0512

**Table 6:** Haplotype analysis of the 3′-UTR SNPs in the *ZDHHC19* gene. *p*: *p*-value of statistical significance of the association between the investigated haplotype and subject cohorts as assessed by *χ*^2^-analysis.

Haplotype rs112579116 rs2293161	Frequency		Frequency		Chi Square	*p*
	1000 Genomes Project	All Patients	Infected Cohort	Septic Cohort		
GC	0.873	0.914	0.882	0.932	1.789	0.1811
GA	0.044	0.057	0.084	0.041	1.952	0.1623
AC	0.083	0.027	0.027	0.027	0.000	0.9921

## Data Availability

The data presented in this study are available upon request from the corresponding author.

## References

[1] Singer M., Deutschman C.S., Seymour C.W., Shankar-Hari M., Annane D., Bauer M., Bellomo R., Bernard G.R., Chiche J.D., Coopersmith C.M. (2016). The Third International Consensus Definitions for Sepsis and Septic Shock (Sepsis-3). JAMA.

[2] Marik P.E., Taeb A.M. (2017). SIRS, qSOFA and new sepsis definition. J. Thorac. Dis..

[3] Rudd K.E., Johnson S.C., Agesa K.M., Shackelford K.A., Tsoi D., Kievlan D.R., Colombara D.V., Ikuta K.S., Kissoon N., Finfer S. (2020). Global, regional, and national sepsis incidence and mortality, 1990-2017: Analysis for the Global Burden of Disease Study. Lancet.

[4] Reyes M., Filbin M.R., Bhattacharyya R.P., Billman K., Eisenhaure T., Hung D.T., Levy B.D., Baron R.M., Blainey P.C., Goldberg M.B. (2020). An immune-cell signature of bacterial sepsis. Nat. Med..

[5] Pinheiro da Silva F., Cataldi T.R., de Lima T.M., Starzynski P.N., Barbeiro H.V., Labate M.T., CéMachado M.C., de Souza H.P., Labate C.A. (2016). Proteomic profiling identifies N-acetylmuramoyl-l-alanine amidase as a novel biomarker of sepsis. Biomark. Med..

[6] Li L., Huang L., Huang C., Xu J., Huang Y., Luo H., Lu X., He S., Yuan G., Chen L. (2022). The multiomics landscape of serum exosomes during the development of sepsis. J. Adv. Res..

[7] Memar M.Y., Baghi H.B. (2019). Presepsin: A promising biomarker for the detection of bacterial infections. Biomed. Pharmacother..

[8] Angeletti S., Spoto S., Fogolari M., Cortigiani M., Fioravanti M., De Florio L., Curcio B., Cavalieri D., Costantino S., Dicuonzo G. (2015). Diagnostic and prognostic role of procalcitonin (PCT) and MR-pro-Adrenomedullin (MR-proADM) in bacterial infections. APMIS.

[9] Paul A., Newbigging N.S., Lenin A., Gowri M., Varghese J.S., Nell A.J., Abhilash K.P.P., Binu A.J., Chandiraseharan V.K., Iyyadurai R. (2023). Role of Neutrophil Gelatinase-associated Lipocalin (NGAL) and Other Clinical Parameters as Predictors of Bacterial Sepsis in Patients Presenting to the Emergency Department with Fever. Indian J. Crit. Care Med..

[10] Song L., Jiang W., Lin H., Yu J., Liu K., Zheng R. (2024). Post-translational modifications in sepsis-induced organ dysfunction: Mechanisms and implications. Front. Immunol..

[11] Binnie A., Tsang J.L.Y., Hu P., Carrasqueiro G., Castelo-Branco P., Dos Santos C.C. (2020). Epigenetics of Sepsis. Crit. Care Med..

[12] Sun Z., Song Y., Li J., Li Y., Yu Y., Wang X. (2023). Potential biomarker for diagnosis and therapy of sepsis: Lactylation. Immun. Inflamm. Dis..

[13] Wu D., Spencer C.B., Ortoga L., Zhang H., Miao C. (2024). Histone lactylation-regulated METTL3 promotes ferroptosis via m6A-modification on ACSL4 in sepsis-associated lung injury. Redox Biol..

[14] An S., Yao Y., Hu H., Wu J., Li J., Li L., Wu J., Sun M., Deng Z., Zhang Y. (2023). PDHA1 hyperacetylation-mediated lactate overproduction promotes sepsis-induced acute kidney injury via Fis1 lactylation. Cell Death Dis..

[15] Mesquita F.S., Abrami L., Linder M.E., Bamji S.X., Dickinson B.C., van der Goot F.G. (2024). Mechanisms and functions of protein S-acylation. Nat. Rev. Mol. Cell Biol..

[16] Jin J., Zhi X., Wang X., Meng D. (2021). Protein palmitoylation and its pathophysiological relevance. J. Cell. Physiol..

[17] Kim Y.C., Lee S.E., Kim S.K., Jang H.D., Hwang I., Jin S., Hong E.B., Jang K.S., Kim H.S. (2019). Toll-like receptor mediated inflammation requires FASN-dependent MYD88 palmitoylation. Nat. Chem. Biol..

[18] Kang J., Wu J., Liu Q., Jiang H., Li W., Li Y., Li X., Ni C., Wu L., Liu M. (2024). FASN regulates STING palmitoylation via malonyl-CoA in macrophages to alleviate sepsis-induced liver injury. Biochim. Biophys. Acta. Mol. Basis Dis..

[19] Zhu X.X., Meng X.Y., Zhang A.Y., Zhao C.Y., Chang C., Chen T.X., Huang Y.B., Xu J.P., Fu X., Cai W.W. (2024). Vaccarin alleviates septic cardiomyopathy by potentiating NLRP3 palmitoylation and inactivation. Phytomed. Int. J. Phytother. Phytopharm..

[20] Donkel S.J., Portilla Fernández E., Ahmad S., Rivadeneira F., van Rooij F.J.A., Ikram M.A., Leebeek F.W.G., de Maat M.P.M., Ghanbari M. (2021). Common and Rare Variants Genetic Association Analysis of Circulating Neutrophil Extracellular Traps. Front. Immunol..

[21] Chen I.C., Chen H.H., Jiang Y.H., Hsiao T.H., Ko T.M., Chao W.C. (2023). Whole transcriptome analysis to explore the impaired immunological features in critically ill elderly patients with sepsis. J. Transl. Med..

[22] He Y.D., Wohlford E.M., Uhle F., Buturovic L., Liesenfeld O., Sweeney T.E. (2021). The Optimization and Biological Significance of a 29-Host-Immune-mRNA Panel for the Diagnosis of Acute Infections and Sepsis. J. Pers. Med..

[23] Li D., Liu Y., Lu Y., Gao S., Zhang L. (2022). Palmitoylation of SARS-CoV-2 S protein is critical for S-mediated syncytia formation and virus entry. J. Med. Virol..

[24] Zhang N., Zhao H., Zhang L. (2019). Fatty Acid Synthase Promotes the Palmitoylation of Chikungunya Virus nsP1. J. Virol..

[25] Tong D.L., Kempsell K.E., Szakmany T., Ball G. (2020). Development of a Bioinformatics Framework for Identification and Validation of Genomic Biomarkers and Key Immunopathology Processes and Controllers in Infectious and Non-infectious Severe Inflammatory Response Syndrome. Front. Immunol..

[26] Baumgart F., Corral-Escariz M., Pérez-Gil J., Rodríguez-Crespo I. (2010). Palmitoylation of R-Ras by human DHHC19, a palmitoyl transferase with a CaaX box. Biochim. Biophys. Acta.

[27] Yhang M., Zhou L., Xu Y., Yang M., Xu Y., Komaniecki G.P., Kosciuk T., Chen X., Lu X., Zou X. (2020). A STAT3 palmitoylation cycle promotes TH17 differentiation and colitis. Nature.

[28] Suire S., Hawkins P., Stephens L. (2002). Activation of phosphoinositide 3-kinase γ by Ras. Curr. Biol. CB.

[29] Liang S., Zhang X., Li J. (2022). Zinc finger Asp-His-His-Cys palmitoyl -acyltransferase 19 accelerates tumor progression through wnt/β-catenin pathway and is upregulated by miR-940 in osteosarcoma. Bioengineered.

[30] Zhang H., Nguyen-Jackson H., Panopoulos A.D., Li H.S., Murray P.J., Watowich S.S. (2010). STAT3 controls myeloid progenitor growth during emergency granulopoiesis. Blood.

[31] Andersen C.L., Jensen J.L., Ørntoft T.F. (2004). Normalization of real-time quantitative reverse transcription-PCR data: A model-based variance estimation approach to identify genes suited for normalization, applied to bladder and colon cancer data sets. Cancer Res..

[32] Korma W., Mihret A., Tarekegn A., Chang Y., Hwang D., Tessema T.S., Lee H. (2020). Identification of Circulating miR-22-3p and miR-93-5p as Stable Endogenous Control in Tuberculosis Study. Diagnostics.

[33] Menyhart O., Weltz B., Győrffy B. (2021). MultipleTesting.com: A tool for life science researchers for multiple hypothesis testing correction. PLoS ONE.

[34] Sticht C., De La Torre C., Parveen A., Gretz N. (2018). miRWalk: An online resource for prediction of microRNA binding sites. PLoS ONE.

[35] Bhattacharya A., Ziebarth J.D., Cui Y. (2014). PolymiRTS Database 3.0: Linking polymorphisms in microRNAs and their target sites with human diseases and biological pathways. Nucleic Acids Res..

[36] Rathour S., Kumar S., Hadda V., Bhalla A., Sharma N., Varma S. (2015). PIRO concept: Staging of sepsis. J. Postgrad. Med..

[37] Zhang T.N., Wen R., Yang Y.H., Yang N., Liu C.F. (2023). Integration of transcriptomic, proteomic, and metabolomic data to identify lncRNA rPvt1 associations in lipopolysaccharide-treated H9C2 cardiomyocytes. Front. Genet..

[38] Middleton E.A., Rowley J.W., Campbell R.A., Grissom C.K., Brown S.M., Beesley S.J., Schwertz H., Kosaka Y., Manne B.K., Krauel K. (2019). Sepsis alters the transcriptional and translational landscape of human and murine platelets. Blood.

[39] Beaumont R., Tang K., Gwee A. (2024). The Sensitivity and Specificity of Procalcitonin in Diagnosing Bacterial Sepsis in Neonates. Hosp. Pediatr..

[40] Langley R.J., Wong H.R. (2017). Early Diagnosis of Sepsis: Is an Integrated Omics Approach the Way Forward?. Mol. Diagn. Ther..

[41] Liu W., Geng F., Yu L. (2020). Long non-coding RNA MALAT1/microRNA 125a axis presents excellent value in discriminating sepsis patients and exhibits positive association with general disease severity, organ injury, inflammation level, and mortality in sepsis patients. J. Clin. Lab. Anal..

[42] Caidengbate S., Akama Y., Banerjee A., Mokmued K., Kawamoto E., Gaowa A., McCullough L.D., Shimaoka M., Lee J., Park E.J. (2023). MicroRNA Profiles in Intestinal Epithelial Cells in a Mouse Model of Sepsis. Cells.

[43] Abdelaleem O.O., Mohammed S.R., El Sayed H.S., Hussein S.K., Ali D.Y., Abdelwahed M.Y., Gaber S.N., Hemeda N.F., El-Hmid R.G.A. (2022). Serum miR-34a-5p and miR-199a-3p as new biomarkers of neonatal sepsis. PLoS ONE.

[44] Sun B., Guo S. (2021). miR-486-5p Serves as a Diagnostic Biomarker for Sepsis and Its Predictive Value for Clinical Outcomes. J. Inflamm. Res..

[45] Zheng G., Qiu G., Ge M., Meng J., Zhang G., Wang J., Huang R., Shu Q., Xu J. (2020). miR-10a in Peripheral Blood Mononuclear Cells Is a Biomarker for Sepsis and Has Anti-Inflammatory Function. Mediat. Inflamm..

[46] Buonsenso D., Sodero G., Valentini P. (2022). Transcript host-RNA signatures to discriminate bacterial and viral infections in febrile children. Pediatr. Res..

[47] Elek Z., Losoncz E., Fülep Z., Kovács-Nagy R., Bánlaki Z., Szlobodnyik G., Keszler G., Rónai Z. (2023). Persistent sepsis-induced transcriptomic signatures in signaling pathways of peripheral blood leukocytes: A pilot study. Hum. Immunol..

[48] Ma S.R., Ma Q., Ma Y.N., Zhou W.J. (2023). Comprehensive analysis of ceRNA network composed of circRNA, miRNA, and mRNA in septic acute kidney injury patients based on RNA-seq. Front. Genet..

[49] Luo X., Lu W., Zhao J., Hu J., Chen E., Fu S., Fu Q. (2022). Identification of the TF-miRNA-mRNA co-regulatory networks involved in sepsis. Funct. Integr. Genom..

[50] Liu S.M., Lin C.H., Lu J., Lin I.Y., Tsai M.S., Chen M.H., Ma N. (2018). miR-596 Modulates Melanoma Growth by Regulating Cell Survival and Death. J. Investig. Dermatol..

[51] Dai J., Yuan G., Li Y., Zhou H. (2021). MicroRNA-596 is epigenetically inactivated and suppresses prostatic cancer cell growth and migration via regulating Wnt/β-catenin signaling. Clin. Transl. Oncol. Off. Publ. Fed. Span. Oncol. Soc. Natl. Cancer Inst. Mex..

[52] Li C., Zheng H., Xiong J., Huang Y., Li H., Jin H., Ai S., Wang Y., Su T., Sun G. (2022). miR-596-3p suppresses brain metastasis of non-small cell lung cancer by modulating YAP1 and IL-8. Cell Death Dis..

[53] Hu X., Zhang J., Bu J., Yang K., Xu S., Pan M., Xiang D., Chen W. (2022). MiR-4733-5p promotes gallbladder carcinoma progression via directly targeting kruppel like factor 7. Bioengineered.

[54] Ohno Y., Kihara A., Sano T., Igarashi Y. (2006). Intracellular localization and tissue-specific distribution of human and yeast DHHC cysteine-rich domain-containing proteins. Biochim. Biophys. Acta.

[55] Xu H., Liu X., Ni H. (2020). Clinical significance of miR-19b-3p in patients with sepsis and its regulatory role in the LPS-induced inflammatory response. Eur. J. Med. Res..

[56] Zhu X. (2020). MiR-125b but not miR-125a is upregulated and exhibits a trend to correlate with enhanced disease severity, inflammation, and increased mortality in sepsis patients. J. Clin. Lab. Anal..

[57] Wang Y., Xu Z., Yue D., Zeng Z., Yuan W., Xu K. (2020). Linkage of lncRNA CRNDE sponging miR-181a-5p with aggravated inflammation underlying sepsis. Innate Immun..

[58] Zheng G., Xiang W., Pan M., Huang Y., Li Z. (2019). Identification of the association between rs41274221 polymorphism in the seed sequence of microRNA-25 and the risk of neonate sepsis. J. Cell. Physiol..

[59] Wang L., Wang H.C., Chen C., Zeng J., Wang Q., Zheng L., Yu H.D. (2013). Differential expression of plasma miR-146a in sepsis patients compared with non-sepsis-SIRS patients. Exp. Ther. Med..

[60] Xiang M., Zeng Y., Yang R., Xu H., Chen Z., Zhong J., Xie H., Xu Y., Zeng X. (2014). U6 is not a suitable endogenous control for the quantification of circulating microRNAs. Biochem. Biophys. Res. Commun..

[61] Dai J., Kumbhare A., Youssef D., Yao Z.Q., McCall C.E., El Gazzar M. (2017). Expression of C/EBPβ in myeloid progenitors during sepsis promotes immunosuppression. Mol. Immunol..

[62] Nystrom G.J., Lang C.H. (2008). Sepsis and AMPK Activation by AICAR Differentially Regulate FoxO-1, -3 and -4 mRNA in Striated Muscle. Int. J. Clin. Exp. Med..

[63] Wang L., Fan H., Sun M., Ye J.H. (2025). SIRT5-mediated HOXA5 desuccinylation inhibits ferroptosis to alleviate sepsis induced-lung injury. Kaohsiung J. Med. Sci..

